# Usefulness of dexmedetomidine to prevent emergence agitation in a patient with Krabbe disease: a case report

**DOI:** 10.1186/s40981-018-0171-4

**Published:** 2018-04-21

**Authors:** Junichi Saito, Futoshi Kimura, Hiroshi Hashimoto, Tetsuhiro Sakai, Kazuyoshi Hirota

**Affiliations:** 1 Department of Anesthesiology, Hirosaki University Graduate School of Medicine, Zaifu-cho 5, Hirosaki, 036-8562 Japan; 2 Department of Anesthesiology, Odate Municipal General Hospital, Odate, Japan; 3 Department of Anesthesiology, Mutsu General Hospital, Mutsu, Japan

**Keywords:** Krabbe disease, Dexmedetomidine, Sleep apnea syndrome, Emergence agitation, Spasticity

## Abstract

**Background:**

We report the case of a child with Krabbe disease who underwent three repeated surgeries and anesthetic management, and we discuss the major concerns about Krabbe disease and the usefulness of a perioperative administration of dexmedetomidine to prevent emergence agitation and hypertension. The patient was scheduled to undergo bilateral orchiopexy, adenotonsillectomy, and knee flexor tendon lengthening under general anesthesia during a 2-year period.

**Case presentation:**

Adenotonsillectomy was scheduled as the second operation when the patient was 4 years old. His height and body weight were 93 cm and 10.3 kg, respectively. Anesthesia was induced with 8% sevoflurane mixed with 6 L/min of O_2_ and maintained with N_2_O (3.5 L/min), O_2_ (1.5 L/min), and sevoflurane (1.5–2.0%). Upon completion of the right tonsillectomy, 1 h before the end of the surgery, a continuous infusion of dexmedetomidine (0.2 μg/kg/h) was started to prevent emergence agitation, irritability, resultant hypertension, and postoperative bleeding. Fentanyl (25 μg) was administered intravenously to reduce postoperative pain. The surgery was uneventful, and the patient’s emergence from general anesthesia was prompt. He exhibited no symptoms of emergence agitation or irritability. During his stay in the intensive care unit, 0.2–0.7 μg/kg/h of dexmedetomidine and 6.25 μg/h of fentanyl were continuously administered. The patient was discharged to the ward the following morning without complications.

**Conclusions:**

The perioperative administration of dexmedetomidine was useful to prevent emergence agitation, hypertension, and resultant postoperative bleeding in a pediatric patient with Krabbe disease. Three repeated anesthetic management using inhalation anesthesia were completed uneventfully without muscle relaxants.

## Background

Krabbe disease is a lysosomal storage disorder caused by galactocerebrosidase deficiency, which leads to the accumulation of psychosine and resultant apoptosis of myelin-forming cells [1]. Patients with Krabbe disease develop progressive neurologic deterioration and death in early childhood; however, the transplantation of umbilical cord blood is associated with substantially better neurologic outcomes and survival [2]. Thus, the anesthetic opportunities for patients with Krabbe disease are expected to increase. Based on the rarity and poor prognosis of this disease, the utility and safety of anesthetic drugs for patients with Krabbe disease are not well established. We herein report the case of a young child with Krabbe disease who underwent three surgeries and anesthetic management, and we discuss the major concerns about Krabbe disease and the usefulness of a perioperative administration of dexmedetomidine to prevent emergence agitation and hypertension.

## Case presentation

A male infant was diagnosed with globoid cell leukodystrophy (Krabbe disease) at birth; his older brother had already been diagnosed with the same disease. Transplantation of umbilical cord blood was performed at 42 days of age. After the transplantation, the patient’s blood galactocerebrosidase concentration improved to a normal value (102 nmol/L; normal range 99 ± 22 nmol/L). However, he had pathognomonic symptoms including irritability, dysphagia, and progressive spasticity. He had no history of seizures. A physical examination revealed spastic paraplegia. He was diagnosed with undescended testis, tonsillar hypertrophy leading to sleep apnea syndrome, and spasticity of the lower extremities.

Bilateral orchiopexy, tonsillectomy and adenoidectomy (T&A), and knee flexor tendon lengthening under general anesthesia were therefore scheduled over a 2-year period. In this report, we focus on the second operation (the T&A) because its perioperative management to prevent emergence agitation and hypertension was more complicated than that during the other two operations.

The T&A was scheduled as the second operation when the patient was 4 years old. Preoperative polysomnography revealed that his sleep apnea was severe: respiratory disturbance index 53.4/min; 3% oxygen desaturation index 46.3/min; nadir SpO_2_ 78% and SpO_2_ < 90% 0.52%. Decreased motor nerve conduction velocity (which reflects demyelination) was revealed: right median nerve 12.7 m/sec and right tibial nerve 11.7 m/sec. His height was 93 cm, and his weight was 10.3 kg. No premedication was administered.

The patient was continuously monitored by electrocardiography, the measurement of peripheral O_2_ saturation, the measurement of the end-tidal concentration of carbon dioxide, monitoring of the body temperature, and train-of-four muscular monitoring. Anesthesia was induced with 8% sevoflurane mixed with 6 L/min of O_2_ through a face mask, with soft cushions placed under the patient’s right shoulder to ensure left lateral decubitus positioning and the prevention of airway obstruction and aspiration. After the induction of anesthesia, a venous line was placed in the forearm and an arterial line was placed in the left radial artery. Endotracheal intubation with a 4.5-mm uncuffed tracheal tube was successful without an administration of a muscle relaxant. Anesthesia was maintained with N_2_O (3.5 L/min), O_2_ (1.5 L/min), and sevoflurane (1.5–2.0%). Local anesthetics (1% lidocaine with epinephrine) were locally infiltrated by the surgeon on the bilateral tonsil.

Upon completion of the right tonsillectomy, 1 h before the end of surgery, a continuous infusion of dexmedetomidine (0.2 μg/kg/h) was started to prevent emergence agitation, irritability, resultant hypertension, and postoperative bleeding. Fentanyl (25 μg) was administered intravenously to reduce postoperative pain. The surgery was uneventful, and the patient’s emergence from general anesthesia was prompt. After the confirmation that the train-of-four monitoring was 100%, the trachea was extubated. The duration of surgery and anesthesia were 87 and 169 min, respectively.

The patient exhibited no symptoms of emergence agitation or irritability. After observation for 50 min in the postanesthesia care unit, the patient was admitted to the intensive care unit (ICU) for observation overnight. During his stay in the ICU, 0.2–0.7 μg/kg/h of dexmedetomidine and 6.25 μg/h of fentanyl were continuously administered. The arterial blood gas analysis revealed no hypercapnia, metabolic acidosis, or electrolyte imbalance. Neither bradycardia nor apnea occurred during the postoperative period. The patient was discharged to the ward the following morning and left the hospital uneventfully on postoperative day 6.

During all three operations, general anesthesia was induced and maintained with sevoflurane with or without N_2_O. No muscle relaxant was administered to facilitate the endotracheal intubation or laryngeal mask airway placement. The surgeries were uneventful, and the emergence from anesthesia was prompt. Pentazocine or fentanyl was administered to reduce postoperative pain. After the patient’s emergence from the anesthesia used in the third surgery, he showed agitation, and additional pentazocine was required; a total of 1.5 mg/kg pentazocine was administered intravenously. The patient left the hospital without anesthetic complications.

### Discussion

This case highlights two clinically important issues. First, the perioperative administration of dexmedetomidine was useful to prevent emergence agitation in a patient with Krabbe disease. One of the anesthetic concerns for patients with Krabbe disease is the development of postoperative analgesia or sedation because such patients often exhibit irritability and poor airway tone with a high risk of aspiration. During the second operation, we chose dexmedetomidine to prevent agitation, hypertension, and resultant postoperative bleeding.

Dexmedetomidine is an α-adrenergic agonist with high α_2_-adrenoceptor selectivity. Pre- and postsynaptic activation of α_2_-adrenoceptors in the locus coeruleus and in the dorsal horn of the spinal cord both inhibit the release of norepinephrine and sympathetic activity and terminate the transmission of pain signals [3]. In addition, the activation of α_2_-adrenoceptors inhibits the release of serotonin in the rostral raphe nucleus, which suppresses arousal and fear [4]. A pediatric patient’s perioperative fear about undergoing surgery and the anxiety of separation from his or her parents often increase the incidence of postoperative anxiety and the amount of opioid needed to control postoperative pain in these patients. The mechanism of action of dexmedetomidine seems to be suitable to prevent emergence agitation.

Due to severe sleep apnea, poor airway tone, and dysphagia, airway management was the most important concern in our patient’s case. Perioperative respiratory complications following a T&A are relatively common and have been described to occur more frequently in children of younger age [5], and lower body weight was reported to be a risk factor for these complications [6, 7]. Additionally, dysphagia associated with poor pharyngeal muscle weakness is a risk factor for aspiration leading to respiratory complications [8]. Our patient thus had a high risk of respiratory complications. Taking these factors into consideration, we considered dexmedetomidine a good choice to prevent agitation and hypertension after our patient’s surgery because of its minimal suppression of respiratory function [9].

Meng et al. [10] found that dexmedetomidine was useful to prevent emergence agitation after tonsillectomy without prolongation of emergence from anesthesia and respiratory depression. Although to the best of our knowledge no studies have evaluated the efficacy of dexmedetomidine in patients with other types of leukodystrophy or Krabbe disease, respiratory depression associated with dexmedetomidine is reportedly less severe than that associated with midazolam in the perioperative period in children [11]. In our case, although a continuous infusion of fentanyl was also required postoperatively in the ICU, a continuous infusion of dexmedetomidine could reduce the required amount of fentanyl and prevent emergence agitation without respiratory complication. Dexmedetomidine can help prevent emergence agitation and control postoperative pain even in patients with Krabbe disease.

The second important clinical issue highlighted by this case is that a patient with Krabbe disease successfully underwent three repeated general anesthetic managements using sevoflurane without complications. Other anesthetic concerns for patients with Krabbe disease are seizures and abnormal responses to anesthetic agents including sedative drugs and neuromuscular relaxants. Inhalation anesthesia is usually selected for pediatric patients. Isoflurane has been used for the maintenance of general anesthesia in children with other types of leukodystrophy [12, 13]. Based on our patient’s case and others, inhalation anesthesia with a small amount of fentanyl and pentazocine can be safely used in patients with Krabbe disease [12, 13]. However, inhalation anesthesia can cause seizures, which are common in patients with leukodystrophy. Propofol can suppress seizures and reduce the risk of agitation and excitation after general anesthesia. Thus, the use of propofol for anesthesia can be an alternative method in these patients [14]. The selection of anesthetic drugs remains controversial; therefore, further clinical studies and case reports of patients with Krabbe disease are required to confirm our findings.

Controlling spasticity is an important way to prevent postoperative pain due to muscle spasm. Pain from a muscle spasm is severe and distressing for cerebral palsy patients with spasticity [15]. Drugs such as baclofen, diazepam, and other benzodiazepines that modulate central triggers of spasticity are usually administered orally. These drugs are agonists at γ-aminobutyric acid receptors, inhibiting the release of neurotransmitters and reducing muscle tone, but drowsiness, ataxia, and lethargy are induced as adverse effects and may result in prolonged sedation and muscle relaxation. On the other hand, clonidine and tizanidine are medications that decrease spasticity through effects on α2-adrenergic receptors [16]. No clinical study has revealed that dexmedetomidine improved spasticity, but considering its mechanism of action, dexmedetomidine has the potential to control spasticity, and it can be an option during the perioperative period.

Additionally, in cerebral palsy patients who often undergo lower limb orthopedic surgery (as in our patient’s third surgery), epidural analgesia is recommended to prevent muscle spasm, which is a spinal reflex initiated by pain [15]. Thus, epidural analgesia can be an option to prevent the severe pain associated with muscle spasms in patients with Krabbe disease. However, these patients usually have a disability and/or poor verbal communication skills, and thus, the detection of pain or adverse events associated with epidural catheterization (especially epidural hematoma) might be delayed, and vigilant observation and comprehensive management are required to prevent such events.

The use of muscle relaxants for patients with Krabbe disease is another concern. We did not administer any muscle relaxants for any of our patient’s three operations because of the risks of hyperkalemia following succinylcholine in patients with neuromuscular disease and the prolongation effect following the administration of non-depolarizing agents. Case reports of patients with other types of leukodystrophy have shown that rocuronium, vecuronium, and atracurium can be safely used without a prolongation effect [8–10]. Thus, if rapid sequence induction and intubation is required in a patient with Krabbe disease, a non-depolarizing muscular agent should be used.

## Conclusions

In summary, we have presented three repeated anesthetic management and major concerns in a pediatric patient with Krabbe disease. The perioperative administration of dexmedetomidine was useful to prevent emergence agitation and hypertension in this patient.
